# Chimpanzees as vulnerable subjects in research

**DOI:** 10.1007/s11017-014-9286-4

**Published:** 2014-03-08

**Authors:** Jane Johnson, Neal D. Barnard

**Affiliations:** 1 Department of Philosophy, Macquarie University, Sydney, NSW 2109 Australia; 2 Physicians Committee for Responsible Medicine, 5100 Wisconsin Avenue, Suite 400, Washington, DC 20016 USA

**Keywords:** Chimpanzee, Vulnerable populations, Medical research, Informed consent, Animal research ethics

## Abstract

Using an approach developed in the context of human bioethics, we argue that chimpanzees in research can be regarded as vulnerable subjects. This vulnerability is primarily due to communication barriers and situational factors—confinement and dependency—that make chimpanzees particularly susceptible to risks of harm and exploitation in experimental settings. In human research, individuals who are deemed vulnerable are accorded special protections. Using conceptual and moral resources developed in the context of research with vulnerable humans, we show how chimpanzees warrant additional safeguards against harm and exploitation paralleling those for human subjects. These safeguards should include empowering third parties to act as surrogate decision makers for chimpanzees, ensuring participant “assent,” and avoiding recruitment of animal subjects based merely on convenience.

## Introduction

Nonhuman primates have long been subjects of research in a range of disciplines and for a wide variety of purposes, but recent findings about the cognitive abilities of chimpanzees, in tandem with research demonstrating their capacity to experience severe psychological harms, challenge the appropriateness of their ongoing participation in medical experimentation. These findings open up the possibility that chimpanzees should be considered as vulnerable subjects in medical research and offered similar protections to those now given to vulnerable human populations.

We begin by outlining why chimpanzees are both inherently and situationally vulnerable in research such that they are at particular risk of harm and exploitation. We then describe the nature of the harms and exploitation they may experience in experimental settings before appealing to concepts from human research ethics to ameliorate these vulnerabilities.

## Why are chimpanzees vulnerable subjects?

Following an extensive examination of the bioethics literature, Wendy Rogers, Catriona Mackenzie, and Susan Dodds formulated an account of vulnerability that overcomes many of the well-acknowledged shortcomings in previous accounts [1]. Although their research concerns human bioethics, there is no prima facie reason why their analysis cannot shed light on the case of chimpanzees [2]. In the following section, we will apply their framework to demonstrate why chimpanzees in research are vulnerable subjects.

According to Rogers, Mackenzie, and Dodds, humans, as biological creatures, share a susceptibility to pain, illness, disease, and death, or what they define as “inherent vulnerability.” Owing to risks in ordinary life, humans are all exposed to this fundamental vulnerability. The kind of vulnerability that is of greater interest when considering research ethics, however, is “more than ordinary vulnerability,” which places certain individuals and groups at greater risk of harm and exploitation in research [1]. Thus, while all humans are inherently vulnerable, some are more vulnerable than others as a result of their life circumstances. This second form of vulnerability is labeled “situational” by Rogers and colleagues. Such vulnerability can fluctuate over time, as circumstances change, and is the product of contextual forces such as economic, social, political, and environmental factors. For example, poverty is an economic factor that can place individuals at greater risk of harm and exploitation in research since their decision to join a study may be influenced by the type of financial payments sometimes offered as compensation for participation.

Like humans, chimpanzees are inherently and situationally vulnerable. Their inherent vulnerability follows, as it does for humans, from their biology—that is, their susceptibility to developing illnesses, contracting diseases, and so on—while situational factors put chimpanzees at increased risk of harm and exploitation in natural and captive settings. In the wild, for instance, they can experience situational vulnerabilities that result, for example, from environmental conditions and interactions with other animals. But even for those in natural settings, human political, regulatory, and legal decisions can influence the situational vulnerability of chimpanzees. For instance, until the 1970s, chimpanzees were taken from their mothers in the wild for use in research. Typically, this resulted in killing or severely disabling mother chimpanzees and disrupting chimpanzee families and communities. Importation of wild-caught chimpanzees was banned under the Convention of International Trade in Endangered Species in 1977 [3], and in 1990, the United States Fish and Wildlife Service upgraded the status of African chimpanzees from threatened to endangered so that trafficking from the wild through other countries could not lead to their importation to the United States [4]. Laws passed in the United States affected the fate of chimpanzees in the wild.

When humans place chimpanzees into captivity or when they breed chimpanzees, they create a dependency in these animals. Chimpanzees become situationally vulnerable to increased risk of harm and exploitation because they are reliant on humans to meet their basic survival needs, in addition to their cognitive, emotional, psychological, and social needs. Understanding the needs of chimpanzees in their dependent situation is complicated by difficulties in communicating with these animals. Although chimpanzees show some understanding of spoken human language and can be trained to communicate through American Sign Language or the use of computer keyboards [5–7], their understanding and communication is not sufficient to ensure, at least at this time, that humans can adequately understand and satisfy the needs of the chimpanzees in their care. Communicative impediments could change in the future to the point where the situational vulnerability of chimpanzees might be lessened. As it stands, however, humans hold considerable power and resources in this dependent situation, and this renders chimpanzees open to exploitation and manipulation.

In the context of research with chimpanzees, the concept of what Rogers and colleagues label “pathogenic” vulnerabilities can be introduced [1]. These vulnerabilities are a subset of situational vulnerabilities that result from morally dysfunctional relationships such as discrimination, injustice, and oppression. In typical laboratory settings, chimpanzees are forced to comply with potentially harmful procedures as a matter of course. This treatment is arguably the result of a discriminatory “speciesism” that illegitimately preferences humans and their interests over chimpanzees on the basis of species membership alone.

Chimpanzees can therefore be considered vulnerable subjects due to their greater than normal susceptibility to harm and exploitation in the context of research. In order to formulate measures to address this vulnerability, we now turn to articulating the nature of the harms and exploitation chimpanzees may be exposed to in research.

## The harms and exploitation experienced by chimpanzees in research

Because of their many similarities with humans, chimpanzees have frequently been used to study infectious diseases such as hepatitis and human immunodeficiency virus (HIV). In addition to the obvious potential harms of infection and its sequelae, chimpanzees in such research studies may also experience harms as a consequence of captivity, transportation, handling, and housing.

Research on infectious disease requires medical tests and procedures, sometimes including repeated biopsies. While some procedures such as phlebotomy (drawing blood via an incision in the vein) are mundane for consenting human adults, they are more taxing for chimpanzees, often involving sedation using darting. Darting involves shooting an individual with a projectile loaded with anesthetic. The shots can be painful and frightening, and multiple darts are sometimes required due to the animal’s desperate defensive maneuvers, thereby extending the period of fear further [8, 9]. Anesthetic interventions typically involve the use of ketamine, which can, with chronic use, cause impairment in memory, executive function, and decreased effectiveness in reducing pain and distress from subsequent procedures [10, 11]. Chimpanzees in research are typically separated from their mothers at a premature age, which may lead to behavioral deficits and maternal incompetence for the offspring and a sense of loss and grief for the mother [12, 13]. Transfer between facilities can be highly stressful due to separation from familiar chimpanzees and human caretakers and may require individual quarantine for anywhere from six weeks to more than six months. It typically involves sedation. In a 2006 case, nine generally healthy chimpanzees were transferred from Ohio State University to Primarily Primates, a facility in Texas. One, a fifteen-year-old male, died of cardiac arrest on the day of arrival, and two others died shortly thereafter [14]. Transfers and introductions into new groups can also result in unpredictable aggressive and injurious behaviors [15, 16].

In the laboratory, animals can be subject to noise, crowding, chemical smells, limited food options, and artificial cycles of lighting [17]. Particularly in infectious disease research, chimpanzees can be confined to cages, greatly diminishing their freedom of movement and their access to mental stimulation, which is highly problematic given their cognitive capacities. Social isolation can lead to increases in abnormal behaviors such as stereotypies (repetitive or compulsive movements or postures) and social withdrawal [18]. While human research participants can be offered information and reassurance that may reduce their anxiety in the research context, these measures are not readily available for nonhuman animals.

The harms chimpanzees experience in research can develop into major behavioral disorders. Hope Ferdowsian and colleagues modified a set of human diagnostic criteria for depression and post-traumatic stress disorder (PTSD) for use with nonhuman primates, finding that criteria for depression were satisfied by 58 % of captive chimpanzees exposed to previous traumatic events, compared with 3 % of those living in the wild. Criteria for PTSD were met by 44 % of captive chimpanzees, but less than 1 % in the wild [19]. It is clear that chimpanzees used in research demonstrate signs of serious disorders that can be traced to their captivity and prior participation in research or to the research environment.

Despite its focus on human research, the 1979 *Belmont Report* of the National Commission for the Protection of Human Subjects of Biomedical and Behavioral Research is an important source for the present discussion. This is due to concerns the report raises regarding justice.

The *Belmont Report* flags the problem of including in human subjects’ research individuals from vulnerable groups (e.g., persons with mental disabilities living in institutions) as potential sources of injustice or exploitation because such individuals may be selected solely for reasons of “administrative convenience” [20]. These individuals are situationally vulnerable by virtue of their dependency and so maybe selected for research on inappropriate grounds. Some groups are also burdened by research participation that is not adequately balanced by, for example, enhanced medical knowledge relevant to that group. Heightened risk of harm and exploitation contributes to heightened vulnerability.

Chimpanzees are exposed to the harms associated with experimentation for which they are rarely intended to be beneficiaries. The bulk of medical research on chimpanzees aims to achieve clinical benefit for human patients; that is, knowledge gained from experiments is intended to benefit individuals from an entirely different species (humans) to the cohort enrolled in research (chimpanzees). Unless the diseases and conditions studied are related to those that occur naturally in chimpanzee populations, or as a result of captivity, the results can have no utility for chimpanzees in captivity or in the wild.^1^

In the section that follows, we argue that some of the situational vulnerability of chimpanzees in research can be ameliorated by using strategies normally applied to vulnerable human subjects.

## Which remedies are available?

It might be argued that remedies for the problems of vulnerability described thus far are not in fact needed to address the vulnerability of chimpanzees in research in the United States, because the National Institutes of Health (NIH) recommendations implemented in 2013 include plans for retiring most federally owned chimpanzees, effectively ending most federally funded chimpanzee research [21, 22]. This decision, however, does not affect federally owned chimpanzees who are not yet scheduled for retirement or those owned by private research entities in the United States. These chimpanzees may still be used as experimental subjects and could therefore benefit from the remedies we propose below.

Respecting autonomy is valued in the context of research with humans as a means of protecting the self-determined interests and well-being of research participants. According to the *Belmont Report*, respecting autonomy involves giving “weight to autonomous persons’ considered opinions and choices” [20, p. 5]. Autonomous individuals are those deemed capable of making appropriately informed decisions about whether to enroll in medical research that may involve harms and whether to continue in such research. Respect for this capacity is formalized through the process of informed consent. Persons with limited or diminished autonomy may be made vulnerable to exploitation, indifference, neglect, or medical error, so their interests may need to be protected by others.

Unlike competent human adults, chimpanzees cannot protect their own interests in research via giving or withholding informed consent to being a research subject, since their capacity to understand information relevant to making such a decision is limited or absent. We propose instead that a form of surrogate consent be developed for research involving chimpanzees. This proposal is not entirely new. It parallels the suggestion made in 2005 by Pascal Gagneux and colleagues that research on great apes should follow ethical principles similar to those applied to human subjects incapable of providing informed consent [23].

We suggest that a surrogate decision maker be empowered to act in a potential chimpanzee subject’s interests, as is done for children where a parent or guardian can assume this role. Such decision makers would be bestowed with the authority to authorize or to refuse experimental participation and to revoke such consent at any stage of the research process if ongoing participation is deemed inappropriate. The surrogate’s approval would be essential for research to proceed and to continue (the latter would require regular monitoring of protocols). These surrogate decision makers would need to be individuals experienced in dealing with chimpanzees, and independent of the institutions undertaking the research in question, so that any decisions made about experimental participation would be, and be seen to be, free and un-coerced.

It is also important to obtain the chimpanzee equivalent of “assent” for research to proceed. In pediatric research this concept refers to the willingness of young children to participate in research in spite of their inability to give informed consent. Assent functions to respect the preferences of children who can partially understand what is demanded of them in research, without being capable of understanding an adequate degree of material information, including risks, consequences, and other ramifications of participation. Children who refuse likely should be excluded from research unless the research offers to provide a therapy unavailable elsewhere. Assent by itself is not sufficient, however. If assent is given, informed consent must still be obtained from the subject’s guardian or legally authorized representative.

Chimpanzees are capable of expressing both assent and dissent. Dissent can be conveyed by behaviors such as resistance, refusal, and displays of fear in the course of being captured, confined, restrained, or manipulated, while assent can be expressed through compliance or willingness, where the former involves merely conforming to what is required and the latter involves greater preparedness to engage in an activity.

In assessing the meaning of refusal or compliance behaviors, however, the context in which they occur needs to be considered. In laboratory research, assent is expressed within a structured institutional setting in which chimpanzees rely on humans (caretakers, laboratory technicians, researchers) for their basic needs and often for their survival. In research with humans, relations of institutional dependency raise concerns that participants might be effectively manipulated or even coerced to join research. Given these dangers a surrogate decision maker would need to make a judgment (in consultation with research personnel) about what the behaviors expressed by chimpanzees actually reflect.

Experiences from some psychological and behavioral studies demonstrate that concepts like assent evidenced through voluntary decision making can be used effectively in the context of research with chimpanzees. For instance, research can be designed so that subjects choose whether to participate and can end participation at any time without penalty. This approach was used in experiments into immediate recall undertaken at Kyoto University [24]. In this research, chimpanzees roam freely through an outdoor facility in which computer screens have been installed for the conduct of cognitive experiments, and the chimpanzees are free to participate or not as they see fit [25]. As a chimpanzee approaches the computer screen, face-recognition software identifies the individual and begins the research task, which typically consists of a series of cognitive tests. The individual is rewarded with food, but can stop at any time and will receive adequate food regardless of participation in the research [26]. Although these measures facilitate a level of voluntariness, they still fall short of the kind of voluntariness deemed appropriate in research with humans as, for example, chimpanzees do not have the option of leaving the environment in which they are captive.

Demands of some scientific research methods may change over time, causing a participant to withdraw consent or assent. For instance, a pharmacokinetic study, once begun, may require measurements to be taken at intervals. The withdrawal of assent would mean the withdrawal from research in these circumstances.

The use of surrogate consent and assent would help address some problems of the vulnerability of chimpanzees in research, though further measures would need to be developed to meet concerns about exploitation. One of us, in conjunction with a collaborator, has examined whether it is conceivable to alter research methodologies with nonhuman animals to be akin to human clinical trials [27]. Ethically justified medical research on humans does not involve exploitation—for example, clinical drug research (phases II and III) attempts to balance possible benefits to patient participants with possible harms. We argue it is possible to mirror this situation with research on nonhuman animals.

On this approach, rather than choosing animals as research subjects on the basis of administrative convenience, their use should be limited to situations in which a number of conditions are satisfied, namely, that the disease or condition being investigated is one that naturally occurs in the study animal; the animal enrolled in the experiment is already afflicted with that disease or condition; and participation in the research offers the chance of benefit (or no more than minimal risk) to the individual participant. These animals are in effect “animal patients” and the deployment of this strategy is now growing in research run from specialist veterinary centers and teaching hospitals [27]. On the animal patient approach, if an animal is suffering from a particular disease or condition and is treated akin to a human patient in a clinical trial, he or she may receive benefits that flow from the experimental treatment received, just as human participants may receive benefits in clinical trials designed to evaluate efficacy. For example, dogs with cancer are sometimes enrolled in oncology research protocols. This research has the potential to deliver therapeutic benefit to dogs with cancer for whom no other effective therapies are available [28].

This is not to deny that some animal patients who make up the control arm of studies may receive no benefit or that some animal patients may be harmed, as human participants in trials may be. The point is that harm is not an inevitable outcome of experimental practice.

There are, however, serious technical challenges to implementing the patient approach in the context of research on chimpanzees, not the least of which is the limited number of chimpanzees who may stand to benefit from research related to their medical conditions. Nonetheless, the concept of vulnerability applies in principle to chimpanzees, and the remedies described herein—assent and surrogate consent measures, and limiting research to situations in which individual participants stand to benefit, or at least to not be significantly harmed—provide potential means for addressing the problems of justification in a morally appropriate manner.

## Conclusion

Attending to similarities between vulnerable humans and chimpanzees—their susceptibility to harm and exploitation—suggests new mechanisms to improve protections for chimpanzees who are subject to use in research. Concepts from human research ethics can be instructively applied to chimpanzees so that *if* these animals continue to be sought for use in research, the measures outlined above (assent, surrogate consent, and avoiding recruitment of animal subjects based simply on convenience) should be extended to them. Of course, another way of dealing with the vulnerability of potential participants in research (and one sometimes deployed in the context of human experimentation) is not to enroll vulnerable individuals in studies at all. In the case of chimpanzees, such a policy could contribute to ending the captivity and dependency of chimpanzees in the experimental domain and the abandonment of the practice of scientific research on these vulnerable animals.

## Footnotes

1 The data may of course be relevant if used to assist animals who have had such diseases or conditions imposed on them by humans.
